# Studying Cell Rolling Trajectories on Asymmetric Receptor Patterns

**DOI:** 10.3791/2640

**Published:** 2011-02-13

**Authors:** Chia-Hua Lee, Suman Bose, Krystyn J. Van Vliet, Jeffrey M. Karp, Rohit Karnik

**Affiliations:** Department of Materials Science and Engineering, MIT - Massachusetts Institute of Technology; Department of Mechanical Engineering, MIT - Massachusetts Institute of Technology; HST Center for Biomedical Engineering and Harvard Stem Cell Institute, Brigham and Women's Hospital and Harvard Medical School

## Abstract

Lateral displacement of cells orthogonal to a flow stream by rolling on asymmetric receptor patterns presents an opportunity for development of new devices for label-free separation and analysis of cells^1^. Such devices may use lateral displacement for continuous-flow separation, or receptor patterns that modulate adhesion to distinguish between different cell phenotypes or levels of receptor expression. Understanding the nature of cell rolling trajectories on receptor-patterned substrates is necessary for engineering of the substrates and design of such devices.

Here, we demonstrate a protocol for studying cell rolling trajectories on asymmetric receptor patterns that support cell rolling adhesion^2^. Well-defined, μm-scale patterns of P-selectin receptors were fabricated using microcontact printing on gold-coated slides that were incorporated in a flow chamber. HL60 cells expressing the PSGL-1 ligand ^3^were flowed across a field of patterned lines and visualized on an inverted bright field microscope. The cells rolled and tracked along the inclined edges of the patterns, resulting in lateral deflection^1^. Each cell typically rolled for a certain distance along the pattern edges (defined as the edge tracking length), detached from the edge, and reattached to a downstream pattern. Although this detachment makes it difficult to track the entire trajectory of a cell from entrance to exit in the flow chamber, particle-tracking software was used to analyze and yield the rolling trajectories of the cells during the time when they were moving on a single receptor-patterned line. The trajectories were then examined to obtain distributions of cell rolling velocities and the edge tracking lengths for each cell for different patterns.

This protocol is useful for quantifying cell rolling trajectories on receptor patterns and relating these to engineering parameters such as pattern angle and shear stress. Such data will be useful for design of microfluidic devices for label-free cell separation and analysis.

**Figure:**
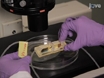


## Protocol

### 1. HL60 cell rolling

#### 1.1. Fabrication of Patterned Substrates.

1. Using microcontact printing (μCP)^4-7^ to make alternating self-assembled monolayers (SAMs) of PEG molecules on the gold-coated glass slides: Fabricate microcontact printing polydimethylsiloxane (PDMS) stamps that defined the receptor patterns with inclination angle of α = 10° by an SU-8 molding process. Clean the gold surface with piranha solution (3:1 mixture of sulfuric acid to 30% hydrogen peroxide) for 20 minutes and then rinse the surface with copious DI water at 24.5 °C prior to use. Ink the PDMS stamp with 5mM PEG solution in ethanol. Dry the stamp in air for 20 minutes. Gently put the stamp on the gold surface for 40 sec and make sure there is a good contact between the gold surface and the stamp. No excess pressure is required. Rinse the surface with ethanol and dry it under a stream of N_2_.
2. Incubate the substrate within P-selectin solution (15 μg/mL in DPBS) using a perfusion chamber (Electron Microscopy Sciences) for 3 hours at 24.5°C to pattern the remaining areas with P-selectin. Rinse the surface with copious DPBS.
3. Backfill the surface with BSA (1 mg/mL in DPBS) for 1 h to block non-specific interactions.  Rinse the surface with copious DPBS.

#### 1.2. Cell Rolling Experiments in a Flow Chamber.

1. Flow a suspension of HL60 cells (~10^5^ cells/mL) over the patterned surfaces in a rectangular flow chamber (Glycotech, Inc; width *w* = 1.0 cm; length = 6 cm; height *h* = 0.0127 cm) at 24.5°C. Use a syringe pump (World Precision Instruments, SP230IW) to generate flow rate of 75 μL/min, with corresponding shear stress around 0.5 dyn/cm^2^ (~0.05 Pa). Calculate shear stress τ by using the plane Poiseuille flow equation τ = *6μQ/wh^2^*, where μ is the kinematic viscosity (0.001002 Pa s), *Q* is volumetric flow rate, *w* is width of the flow chamber, and *h* is height of the flow chamber.
2. Use an inverted microscope (Nikon TE2000-U) with a mounted camera (Andor iXon 885) to record HL60 cells rolling interactions with adhesive P-selectin substrates using a 4× objective, typically at a rate of 1 frame per second for durations of 300 s. Perform three independent experiments, for each shear stress magnitude and pattern inclination angle. Present data as mean and standard deviation of the average values obtained from each experiment.
3. Data Analysis. Analyze the image sequences by a customized Matlab (Mathworks, Inc.) program that utilized a particle tracking freeware^8^ to generate tracks along the patterned line edges. Tracks extending till the end of a P-selectin band are selected and fitted with two straight line segments - one aligned with the flow, the other aligned with the pattern edge. These two segments are then used to calculate the edge tracking length, rolling velocity on the edge, and rolling velocity on the plain region.

### 2. Representative Results:


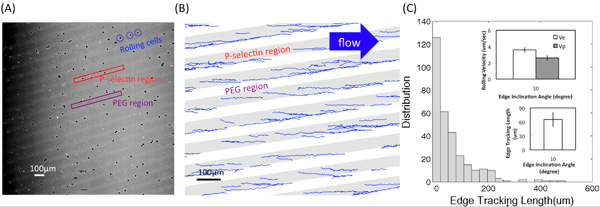
 **Figure (A)** shows one of microscope images converted from the video of HL60 rolling interactions with adhesive P-selectin substrates using a 4× objective. Bright and dark regions correspond to P-selectin receptor and PEG regions, respectively. **Figure (B)** shows the tracks obtained using a customized Matlab program. The edge inclination angle was 10° and the shear stress was 0.5 dyn/cm^2^. The edge tracking length, *l_e_*, displacement, d, and the rolling velocities on the edge and inside the bands, *V_e_* and *V_p_*, respectively, are described in **Figure (C-1)**. **Figure (C-2)** shows the distribution (the number of recorded cells) of edge tracking length. Insets show the average value of *l_e_* and the rolling velocity on the edge (*V_e_*) and inside the bands (*V_p_*) at the inclination angle α =10° and the fluid shear stress magnitude around 0.5 dyn/cm^2^. Error bars represent one standard deviation, where n = 3 replicate experiments (3 replicate surfaces) for each condition.

## Discussion

We have described a protocol to examine cell rolling trajectories on asymmetric receptor-patterned surfaces fabricated using microcontact printing^2^. The optical microscope images of patterned surface showing clear contrast between PEG and P-selectin areas can be used to confirm whether stamping is successful. Sharp, straight edges can be observed when the stamping is performed well. Hard pressing of the stamp may result in stamp deformation which limits the precision of patterns. Wavy edges may be obtained when the ink concentration is too high or the stamping time is too long. Bad contact between the stamp and the surface leads to non-uniform PEG patterns when the size of the stamp is too small (<0.5 cm^2^). Non-fresh ink solution may result in cells rolling on the PEG-patterned regions because of decreased ability to passivate P-selectin. The actual inclination angle can be calculated from images of the pattern and trajectories of free-flowing cells that are not interacting with the surfaces. The experiment described here yields information about individual cell rolling events along pattern edges; however, it is difficult to automatically track cells that detach on one pattern and re-attach on another pattern. Changing the pattern spacing, for example, may enable tracking of a single cell encountering multiple edges. This ability may enable higher resolution for distinguishing between cells with different rolling characteristics at the single cell level. As cell rolling behavior depends on the ligands on the cell, we hypothesize that such experiments may enable design of appropriate patterns to separate different cell phenotypes based on differences in their rolling behavior^3, 9-12^. This work can thus be useful for design of devices for cell separation and analysis based on interaction between cell ligands and the patterned asymmetric receptors.

## Disclosures

No conflicts of interest declared.
